# A Rare Case of Complex Pelvic Injury and Associated Intrathecal Fat Embolism due to Spinopelvic Dislocation with Sacral Burst Fracture

**DOI:** 10.1155/2020/5152179

**Published:** 2020-12-02

**Authors:** Jan-Dierk Clausen, Karsten Fink, Michaela Wilhelmi, Christian Macke, Marcel Winkelmann, Christian Krettek, Philipp Mommsen

**Affiliations:** Trauma Department, Hannover Medical School, Hannover, Germany

## Abstract

**Introduction:**

Pelvic and lumbar spine injuries are very common especially in multiple trauma patients. The usual mechanism in young patients leading to pelvic fractures is a high-energy trauma such as traffic accidents. In elderly patients, low energy traumas are causal for such injuries. Compared to the high number of patients with pelvic or lumbar spine injuries, cerebral fat embolism is a quite rare finding but it needs to be considered to not misinterpret the radiological findings.

**Case:**

We present the case of a 41-year-old patient, who got hit and trapped in the lumbar region by a hydraulic arm in a car repair shop. The patient was primarily admitted to a level II trauma center. The radiological and clinical examinations revealed an open pelvic type C injury in terms of a spinopelvic dissociation, dislocation of the left hip joint, rupture of the mesentery of the rectum and colon sigmoideum, and a complex injury to the left ureter. Additionally, CT scan showed fluid with higher density than cerebro spinal fluid (CSF) in the lateral ventricles indicating an intracranial bleeding. After an immediate surgery to stabilize the patient, he was admitted to a level I trauma center. The reanalysis of the existing CT datasets combined with a new head CT leads to the conclusion that the high density fluid in the lateral ventricles is not a intracranial bleeding but rather fat deriving from the complex pelvic and lumbar spine fracture into the CSF system. Therefore, an immediate operation was performed to stabilize the spinopelvic dissociation and to close the injured dural sheath. Additionally, a ventricle drainage has been placed, which confirmed the diagnosis of intrathecal fat embolism. Afterwards, complex plastic surgery was necessary to restore the soft tissue damage.

**Conclusions:**

Intrathecal fat embolism in muliple trauma patients is a rare condition, which should be considered in patients with complex spine or pelvic injuries. It is important to distinguish this rare condition from intracranial bleedings, which are much more common because the consequent therapeutic strategy is quite different. In case of intrathecal fat embolism, a ventricle drainage system should be placed immediately, and the underlying spine or pelvic injuries need to be stabilized combined with closure of the dural sheath to prevent continuous fat embolism and meningeal infection.

## 1. Introduction

Intrathecal fat embolism is a condition, which is normally seen in patients with long bone fractures [1–3]. In these cases, the fat migrates through the vessels into the brain. The observation of fat droplets in the ventricular system can rarely be found in the existing literature. It has been mostly described in cases of ruptures of intracranial or intraspinal dermoid or tarlov cysts [2, 4, 5], ruptures of teratomas [6, 7], and rarely after removal of intracranial neoplasms such as meningioma [2]. There are also several reports that intracranial fat may occur after aortic valve surgeries [8]. The association of intrathecal fat embolism in the ventricular system after trauma has only been reported a few times and mostly in case reports. Nevertheless, intrathecal fat embolism after spinopelvic injuries needs to be considered. The pelvic ring can be exposed to high forces up to 5800 N without losing its integrity [9]. Nevertheless, pelvic trauma is commonly seen in multiple injured patients. In Germany, around 15% of multiple trauma patients with an Injury Severity Score (ISS) > 15 suffer from pelvic injury. Spine injuries occur in around 30% of multiple injured patients according to the German Trauma Registry. The treatment of unstable pelvic fractures requires high surgical expertise. Common problems are severe hemorrhage and associated intra- or retroperitoneal injuries. The presence of intrathecal fat embolism complicates it even more as immediate definitive stabilization along with closure of the dural sheath is necessary to prevent ongoing embolism, meningeal infection, and further brain damage. The present case is the first describing intrathecal fat embolism in case of the open pelvic fracture.

## 2. Case Scenario

We present the case of a 41-year-old male, who got hit by a moving hydraulic arm in the lumbar region and was pressed against a wall and got stuck in this position. After being freed, he was presented to the emergency room (ER) of the next reachable trauma center which in this case was a level II local trauma center. The clinical examination and the CT scan revealed a type C open pelvic fracture combined with a spinopelvic dissociation and a rupture of the left ureter as well as severe trauma to rectum and colon sigmoideum with a traumatic rupture of the mesenterium. Additionally, a dorsal hip dislocation on the left side and complex bilateral ankle fractures were found.

The patient was suffering from a severe hemorrhage caused by the mesenterial injury making an immediate surgery necessary. The urgent surgical treatment consisted of (1) application of a supracetabular external fixator, (2) debridement of the perineal wound and the open pelvic fracture combined with a vacuseal dressing, (3) splinting of the ureter, and (4) modified Hartmann's procedure addressing the mesenterial and large bowel injuries combined with an perihepatic and perisplenic packing. During operation, the patient was still unstable despite transfusion of 11 packed red blood cells (PRBC), 8 fresh frozen plasma (FFP), and 2.000 IU of fibrinogen. Afterwards. the patient was admitted to the interdisciplinary intensive care unit (ICU). The patient was secondarily transferred to a level I trauma center two days after trauma. At the time point of admission at the emergency room of our level I trauma center, the patient was mechanically ventilated and hemodynamically unstable with high doses of norepinephrine (rate 21 ml/h of 5 mg/50 ml perfusor).

The initial radiographs were analyzed revealing the following diagnoses:

- III° open type C pelvic injury (transpubic and transiliosacral on the right side, transforaminal on the left side)
- Spinopelvic dislocation (S2/3) with a dural tear and intrathecal fat embolism
- Rupture of the left ureter
- Rupture of the mesenterium of the colon sigmoideum and terminal parts of the ileum

**Figure 1:**
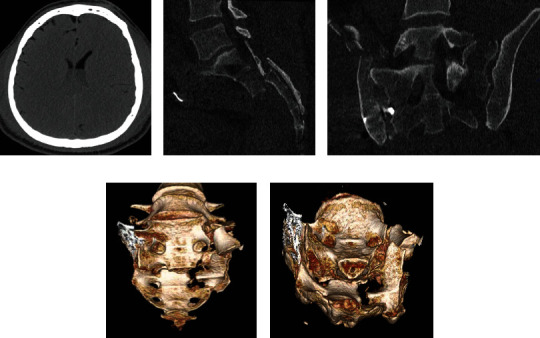
(a) Axial head CT scan indicating hyperdense fluid in the lateral ventricles. (b, c) Sagittal and coronal CT planes' indication on the spinopelvic dislocation and the unstable dorsal pelvic ring with a transiliosacral fracture on the right side and a transforaminal component on the left side. (d, e) 3D reconstruction of the CT scan showing the spinopelvic dislocation and sacral burst fracture (S2/3).

Afterwards, a multiple slice CT (MSCT) was repeated. It revealed a hyperdense fluid collection in the lateral and dorsal ventricles (Figure 1(a)). The consultation of neurosurgery and neuroradiology leads to the diagnosis of intrathecal fat embolism rather than a bleeding. The CT scan of the pelvis showed a persistent spinopelvic dislocation with the sacral burst fracture suggesting a dural tear as cause of the intrathecal fat embolism (Figures 1(b)–1(e)).

**Figure 2:**
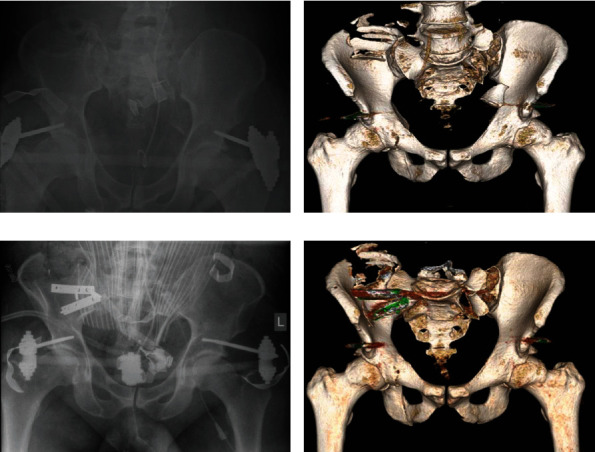
(a) Anteroposterior X-ray of the pelvis at time of administration. (b) 3D-CT reconstruction indicating the instability of the dorsal pelvic ring with a gaping right SI joint. (c) Postoperative anteroposterior X-ray and corresponding 3D-CT reconstruction (d) after plating of the right SI joint and modification of the supracetabular external fixator.

Due to the persistent hemodynamic instability and the remaining biomechanical instability of the dorsal pelvic ring (Figures 2(a) and 2(b)), we performed an immediate surgery with plating of the right SI joint and modification of the supraacetabular external fixator (Figures 2(c) and 2(d)). Afterwards, the situation improved tremendously, and the patient was transferred to the ICU.

The interdisciplinary evaluation of the case resulted in the strategy of early stabilization with reduction of the spinopelvic dissociation and repair of the dural tear and implementation of a ventricular drainage system. 12 hours after admission to the ICU, the patient was physiologically stabilized, and the catecholamine doses decreased to a tolerable situation (rate 5 ml/h of 5 mg/50 ml perfusor). Therefore, surgical treatment was initiated.

**Figure 3:**
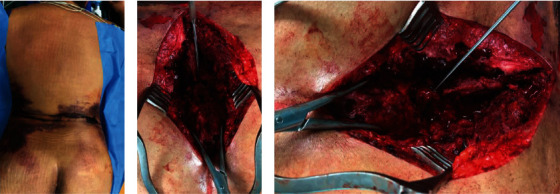
(a) Patient in the prone position with large hematoma indicating the Morel-Lavallée lesion. (b, c) Intraoperative pictures showing the sacral dural tear.

First of all, a ventricular drainage was inserted by the neurosurgeon in a supine position using a computer-based navigation software. The drainage extracted greasy CSF concordant with the radiological findings of intrathecal fat embolism. The erythrocyte count indicated no traumatic intraventricular bleeding. The intracthecal pressure was measured since then during the whole procedure. The patient was then transferred to a prone position. The soft tissue showed serious contusion marks as shown in Figure 3(a). A median incision was made. According to the contusion marks, a closed degloving injury pattern was found in terms of a Morel-Lavallée lesion. Further, preparation revealed a total sacral rupture of the thecal sack. The sacrum was dorsally dislocated between S2 and S3. The spinopelvic complex was completely unstable in coronal, horizontal, and sagittal plane (Figures 3(b) and 3(c)).

A lumbopelvic stabilization was performed L4/5 to iliac bone (os ilium). The sacrum was reduced and fixed with two bilateral 3.5 mm reconstruction plates. Afterwards, a long-distance decompression (sacrotomy) was performed by neurosurgery. The thecal sack was closed in the microsurgical technique and protected by an additional grid plate, which was fixed with 1.2 mm screws.

**Figure 4:**
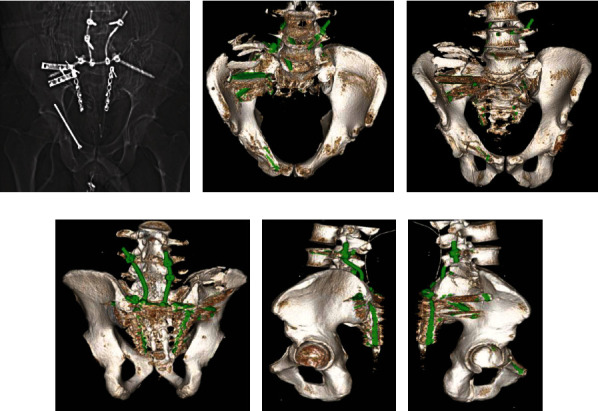
Postoperative radiological analysis. (a) Anteroposterior X-ray of the pelvis. (b)– (f) 3D-CT reconstruction showing the adequate reduction by lumbopelvic stabilization and the anterior acetabular column screw.

**Figure 5:**
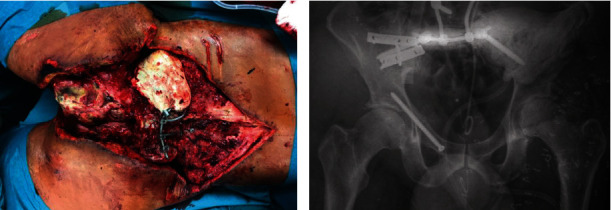
(a) Intraoperative situation after partial resection of the left iliac bone. (b) Anteroposterior X-ray of the pelvis with the bone cement spacer in place.

The postoperative CT scan showed an adequate reduction of the sacrum as well as all implants in the right position (Figures 4(a)–4(f)). The postoperative microbiological analysis revealed a mixed microbiologic flora with detection of Enterococcus faecalis, Enterococcus faecium, Staphylococcus epidermidis, and Candida parapsilosis. Therefore, the patient underwent a debridement with a partial resection of the left iliac bone and sacral implant removal due to an ongoing osteomyelitis. The lateral iliac screw was stabilized by a reconstruction of the os ilium with bone cement containing both gentamicin and vancomycin (Figures 5(a) and 5(b)).

**Figure 6:**
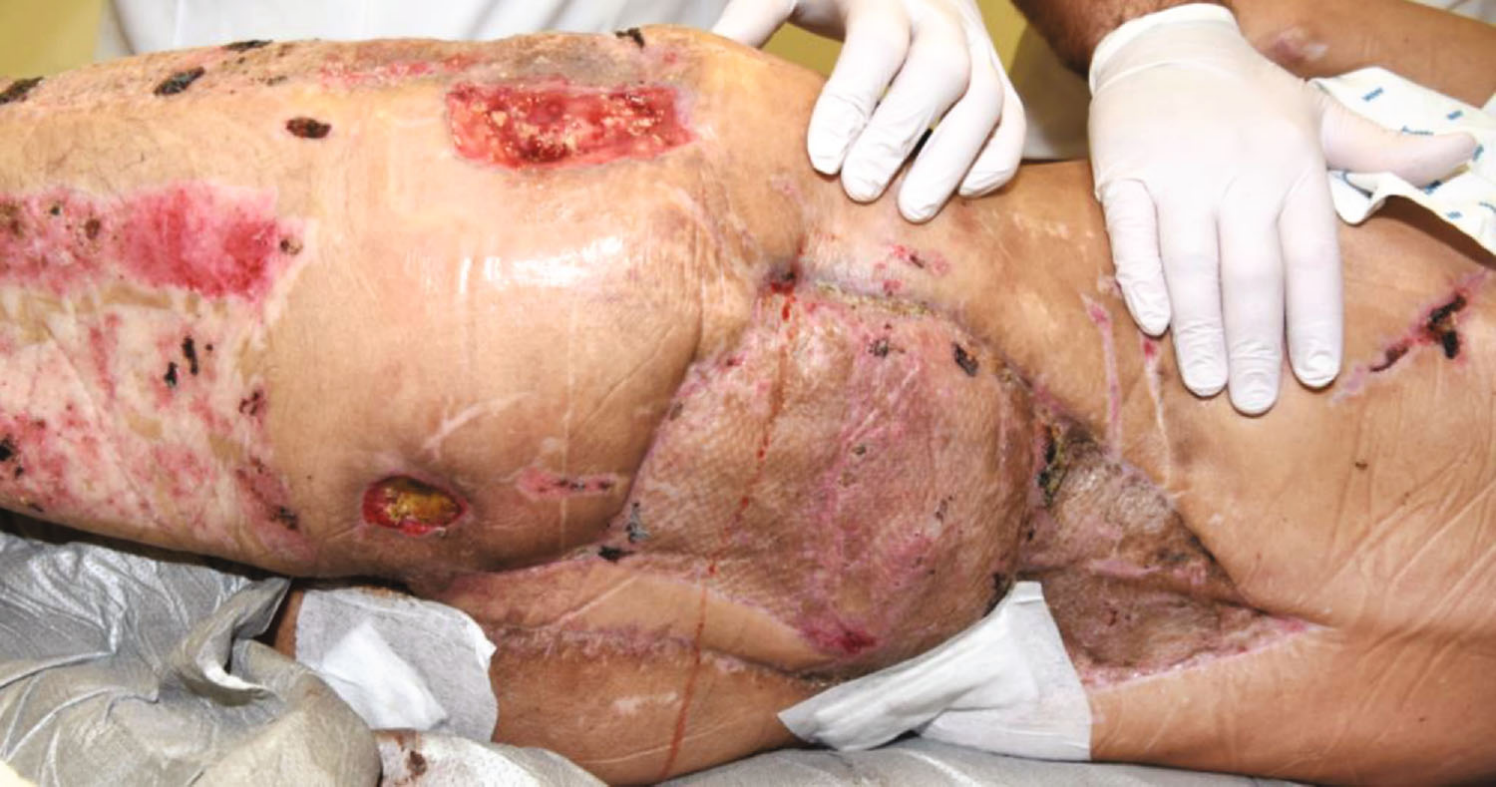
Clinical situation 9 months posttrauma showing the fully healed free muscle flap and skin graft.

In an interdisciplinary discussion with plastic surgeons, the decision was made to strive for a septic wound closure with a free latissimus dorsi flap and skin graft due to the fact that the patient already underwent several debridements, and the chance for complete germ eradication was estimated to be very low. The flap and skin autograft procedure were performed, which both healed in properly (Figure 6).

Overtime, the patient was stabilized, and a tracheotomy was performed. This made it possible to reduce the sedation, and so the patient was finally awake. The neurological status of the patient revealed a complete palsy beneath L3.

Due to disturbed CSF flow, a ventriculoperitoneal shunt was established by neurosurgery in the course of time. The physiotherapist was able to improve the level of mobility. At the time of discharge into a rehabilitation facility, the patient could be mobilized into an upright position in his bed.

## 3. Discussion

Intrathecal fat embolism is a rare condition especially compared to the amount of severe head trauma with intrathecal bleeding. According to the German Trauma Registry, almost 45% of multiple injured patients are suffering from severe head trauma (AIS > 2). Although it is uncommon, intrathecal fat embolism should be kept in mind as a differential diagnosis not only in patients with long bone fractures. Possible injuries with an increased risk for intrathecal fat embolism are complex spinal or pelvic trauma. Due to its rarity, there are no absolute or relative numbers of the actual risk for intrathecal fat embolism following this kind of trauma.

However, especially physicians working in a level I trauma center should be familiar with this particular pathology. The treatment plan should according to the ATLS criteria first focus on the initial patient stabilization. Afterwards, the ventricular system should be drained early by applying a ventricular drainage. In order to prevent further embolism and ingress of microbes, fixing the thecal sack and stabilizing unstable spine and/or pelvic injuries should be done as soon as possible.

If the drained cerebral fluid stays greasy or a disturbance of the CSF flow occurs in the further time course, a definitive drainage (e.g., ventriculoperitoneal shunt) should be installed.

## Data Availability

There are no datasets due to the fact that the manuscript represents a case report.

## References

[1] Simon A. D., Ulmer J. L., Strottmann J. M. (2003). Contrast-enhanced MR imaging of cerebral fat embolism: case report and review of the literature. *AJNR. American Journal of Neuroradiology*.

[2] Woo J. K. H., Malfair D., Vertinsky T., Heran M. K. S., Graeb D. (2010). Intracranial transthecal subarachnoid fat emboli and subarachnoid haemorrhage arising from a sacral fracture and dural tear. *The British Journal of Radiology*.

[3] Moliere S., Kremer S., Bierry G. (2018). Case 254: posttraumatic migrating fat embolus causing fat emboli syndrome. *Radiology*.

[4] Smith A. S., Benson J. E., Blaser S. I., Mizushima A., Tarr R. W., Bellon E. M. (1991). Diagnosis of ruptured intracranial dermoid cyst: value MR over CT. *AJNR. American Journal of Neuroradiology*.

[5] Duja C. M., Berna C., Kremer S., Géronimus C., Kopferschmitt J., Bilbault P. (2010). Confusion after spine injury: cerebral fat embolism after traumatic rupture of a Tarlov cyst: case report. *BMC Emergency Medicine*.

[6] Bhangoo R. S., Tammam A., Crockard H. A. (1997). MRI detection of spontaneous rupture of a well differentiated pineal teratoma. *Acta Neurochirurgica*.

[7] Harrison R. L., Abernethy L. J. (2001). Asymptomatic intraventricular lipid leak from a primary pineal teratoma. *Pediatric Radiology*.

[8] Lee T. C., Bartlett E. S., Fox A. J., Symons S. P. (2005). The hypodense artery sign. *AJNR. American Journal of Neuroradiology*.

[9] Winkelmann M., Lopez Izquierdo M., Clausen J. D. (2018). Fractures of the transverse processes of the fourth and fifth lumbar vertebrae in patients with pelvic ring injuries: indicator of biomechanical instability but not shock. *The Bone & Joint Journal*.

